# Generation of Self-Induced Myocardial Ischemia in Large-Sized Cardiac Spheroids without Alteration of Environmental Conditions Recreates Fibrotic Remodeling and Tissue Stiffening Revealed by Constriction Assays

**DOI:** 10.1021/acsbiomaterials.3c01302

**Published:** 2024-01-18

**Authors:** Laura Paz-Artigas, Sandra González-Lana, Nicolás Polo, Pedro Vicente, Pilar Montero-Calle, Miguel A. Martínez, Gregorio Rábago, Margarida Serra, Felipe Prósper, Manuel M. Mazo, Arantxa González, Ignacio Ochoa, Jesús Ciriza

**Affiliations:** † Tissue Microenvironment (TME) Lab, Aragón Institute of Engineering Research (I3A), University of Zaragoza, Zaragoza 50018, Spain; ‡ Institute for Health Research Aragón (IIS Aragón), Zaragoza 50009, Spain; § BEONCHIP S.L., CEMINEM, Campus Río Ebro, Zaragoza 50018, Spain; ∥ Instituto de Biologia Experimental e Tecnológica (iBET), Oeiras 2780-157, Portugal; ⊥ Instituto de Tecnologia Química e Biológica António Xavier, Universidade Nova de Lisboa, Oeiras 2780-157, Portugal; # CIBER-BBN, ISCIII, Zaragoza 50018, Spain; ¶ Cardiology and Cardiac Surgery Department, Clínica Universidad de Navarra, Pamplona 31009, Spain; ∇ Regenerative Medicine Program, Cima Universidad de Navarra, and Instituto de Investigación Sanitaria de Navarra (IdiSNA), Pamplona 31008, Spain; ○ Hematology and Cell Therapy, Clínica Universidad de Navarra, and Instituto de Investigación Sanitaria de Navarra (IdiSNA), Pamplona 31008, Spain; ⧫ CIBERONC, Instituto de Salud Carlos III, Madrid 28029, Spain; †† Program of Cardiovascular Diseases, CIMA Universidad de Navarra, and Instituto de Investigación Sanitaria de Navarra (IdiSNA), Pamplona 31008, Spain; ‡‡ CIBERCV, Instituto de Salud Carlos III, Madrid 28029, Spain

**Keywords:** myocardial ischemia, cardiac spheroid, fibrosis, hiPCS-CM, stiffness

## Abstract

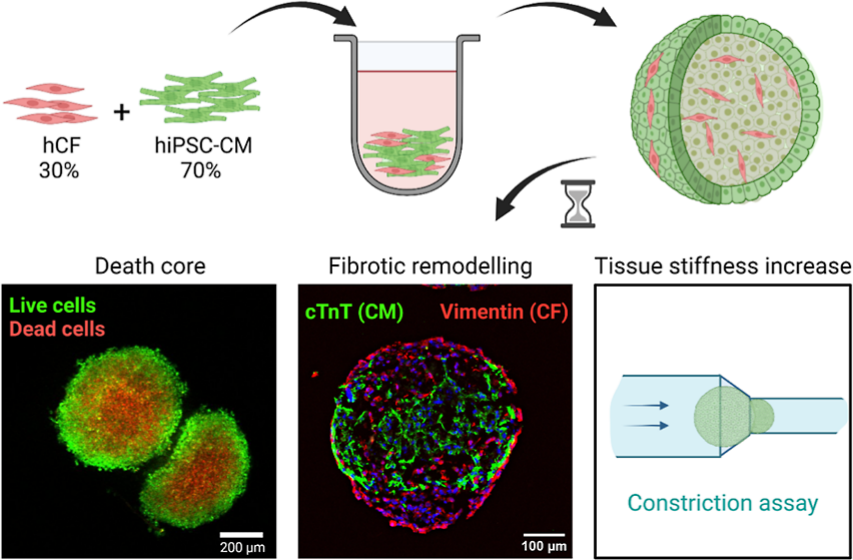

A combination of human-induced pluripotent stem cells (hiPSCs) and 3D microtissue culture techniques allows the generation of models that recapitulate the cardiac microenvironment for preclinical research of new treatments. In particular, spheroids represent the simplest approach to culture cells in 3D and generate gradients of cellular access to the media, mimicking the effects of an ischemic event. However, previous models required incubation under low oxygen conditions or deprived nutrient media to recreate ischemia. Here, we describe the generation of large spheroids (i.e., larger than 500 μm diameter) that self-induce an ischemic core. Spheroids were generated by coculture of cardiomyocytes derived from hiPSCs (hiPSC-CMs) and primary human cardiac fibroblast (hCF). In the proper medium, cells formed aggregates that generated an ischemic core 2 days after seeding. Spheroids also showed spontaneous cellular reorganization after 10 days, with hiPSC-CMs located at the center and surrounded by hCFs. This led to an increase in microtissue stiffness, characterized by the implementation of a constriction assay. All in all, these phenomena are hints of the fibrotic tissue remodeling secondary to a cardiac ischemic event, thus demonstrating the suitability of these spheroids for the modeling of human cardiac ischemia and its potential application for new treatments and drug research.

## Introduction

Cardiovascular diseases have increased in the last decades as a result of population growth and aging, remaining the leading cause of death worldwide in 2019 and a major contributor to reduced quality of life.^1^ Almost half of the cardiovascular disease burden is due to ischemic heart diseases, a set of clinical syndromes characterized by the imbalance between the myocardial blood demand and supply, usually caused by the occlusion of a coronary blood vessel. Preclinical research has benefited from human-induced pluripotent stem cells (hiPSCs) and 3D microtissue culture techniques to recapitulate *in vitro* the complex microenvironment of physiological and pathological human myocardium (*i.e.*, structure, different cell types, cell-to-cell, and cell-to-matrix connections). Despite the recent blossom of these cardiac models,^2,3^ few works have focused on reproducing myocardial ischemia.^4^

Tissue damage during cardiac ischemia tends to be related to an insufficient oxygen supply, but it is also due to a reduced availability of nutrients and inadequate removal of metabolic end-products. In fact, diseases only caused by hypoxia, like cyanosis, severe anemia, or lung diseases, cause less notorious effects than ischemia.^5^ Therefore, modeling an ischemic myocardium *in vitro* requires the recreation of its particular 3D architecture, closely related to the formation of nutrient, oxygen, and cellular product concentration gradients, and generating a spatial increase in cellular damage. Spheroids represent the simplest approach to culture cells in 3D and generate gradients of cellular access to the media. Aggregates of cardiomyocytes (CMs) derived from hiPSC (hiPSC-CMs) have been subjected to reduced nutrient media and hypoxic atmosphere, followed by standard conditions’ restoration to simulate the effects of blood flow restoration.^6^ After such an ischemia/reperfusion protocol, the spheroid core presented lacunae and apoptotic cells. hiPSC-CM also showed disrupted sarcomere myofilaments, mitochondria alterations, and increase secretion of inflammatory, angiogenic, and migration-related molecules.

While CMs represent the most notorious cells in the heart and are most sensible to oxygen and nutrient deprivation, a model of cardiac ischemia also requires the presence of other cell types involved in the cardiac response to ischemia. In particular, cardiac fibroblasts play a key role in ischemic myocardial remodeling. Thus, following ischemic cardiomyocyte death, fibroblasts are recruited to the injured area and activated into myofibroblasts, which synthesize collagen, forming a scar that increases tissue stiffness and further reduces cardiac pumping capacity.^7^ To generate a more complex model of cardiac ischemia, hiPSC-CM have been cocultured in spheroids with human cardiac fibroblasts (hCFs), along with human adipose-derived stem cells and human umbilical vein endothelial cells.^8^ When these complex spheroids were subjected to partial oxygen reduction and adrenergic stimulation, they recreated features of cardiac fibrosis, including cellular reorganization, with hiPSC-CMs located at the core and surrounded by hCFs, and tissue stiffening.^9^ Although several characteristics of ischemic myocardium have been successfully recreated within spheroids, the few reported models of cardiac ischemia worked with relatively small aggregates, requiring incubation in low oxygen conditions or deprived nutrient media to achieve an ischemic core.^6,8,9^

Here, we hypothesized that culturing larger cardiac spheroids that self-induce the ischemic core could more physiologically recapitulate the *in vivo* environment of the ischemic human myocardium, including effects of waste products accumulation. For this purpose, we generated spheroids of at least 500 μm diameter by coculturing hiPSC-CM and primary hCF. We cultured the resulting spheroids for up to 17 days, studying cellular viability and hints of fibrotic remodeling.

## Materials and Methods

### Cell Lines

hCFs, isolated from discarded cardiac surgical tissue from patients undergoing cardiac surgery through explant outgrowth, were routinely cultured with low glucose DMEM (Biowest, France) supplemented with 10% FBS, 5% penicillin/streptomycin, 10 ng/mL fibroblast growth factor 2 (FGF-2) (Miltenyi Biotec, Germany), and 3 μM glutamine.

Human iPSCs (CBiPSsv-4F-40 line) were cultured on 1:80 Growth Factor Reduced-Matrigel (Corning, United States)-coated plastic surfaces in mTeSR1 complete medium (STEMCELL Technologies, Canada) and passaged every 4–5 days through cell detaching by incubation with 0.5 mM EDTA (Invitrogen, United States).

### hiPSC Differentiation into CM

Human iPSC differentiation into CMs was performed following a biphasic Wnt modulation protocol^10^ with minor modifications. After reaching 90% confluence, cells were incubated for 24 h in RPMI (Gibco, United States) supplemented with B27 1× minus insulin (Gibco, United States) (RPMI B27-) and 12 μM CHIR99021 (STEMCELL Technologies, Canada), followed by 48 h of incubation in RPMI B27-. Then, the medium was changed to RMPI B27- supplemented with 5 μM C59 (Sigma-Aldrich, Spain) for 48 h, followed by another 48 h of incubation in RMPI B27-. The medium was then changed to RPMI supplemented with B27 1× and insulin (Gibco, United States) (RPMI B27+) and refreshed every 48 h. Once generalized cellular beating was observed, cells were subjected to 2 purification cycles, consisting of incubation in RPMI without glucose supplemented with 4 mM lactate (Sigma-Aldrich, Spain) for 48 h. After purification, the medium was changed to RPMI B27+ and refreshed every 2 days until hiPSC-CM usage.

For quality control analysis of hiPSC-CM differentiation, the percentage of cells expressing the cardiac marker troponin was quantified by flow cytometry (Figure S1). Briefly, cells from random wells were detached using TryplE and processed using a FIX & PERM cell permeabilization kit (Invitrogen, United States), following the manufacturer’s instructions. Cells were stained with a cardiac Troponin T (cTnT) Monoclonal Antibody from mouse (clone 13-11, MA5-12,960, Invitrogen, 1:100), incubated for 30 min at RT, followed by a secondary antibody Alexa Fluor 488 goat antimouse (A-11001, Invitrogen, 1:100) for 15 min RT in the dark. All samples were then centrifuged in 2 mL of FACS buffer for 5 min at 1800 rpm 3 times. Each pellet was resuspended in PBS and stored at 4 °C until analysis. Differentiation batches with less than 70% of cTnT+ cells were discarded.

### Cell Metabolic Activity

For the metabolic activity characterization of hCF and hiPSC-CM cells in different media compositions, both cell types were separately seeded in 96-well plates, with hCF maintenance medium and RPMI (plus B27+ and 5 μM Y-27632) medium for 24 h, respectively. The medium was changed to the tested medium, and the solution was reused every 2 days. Tested media consisted of (1) hCF basal medium composed of low glucose DMEM supplemented with 3 μM glutamine and 0.5 ng/mL FGF-2; (2) hiPSC-CM basal medium composed of RPMI medium supplemented with B27 1× and insulin; (3) hCF basal medium supplemented with B27 1× and insulin; (4) hiPSC-CM basal medium supplemented with 0.5 ng/mL FGF-2, and (5) a 50:50 mixture of hCF and hiPSC-CM basal media.

After 7 days, metabolic activity was assessed by 3-[4,5-dimethylthiazol-2-yl]-2,5-diphenyl tetrazolium bromide (MTT) metabolic assay (Sigma-Aldrich, Spain), following the fabricant’s recommendations. Absorbance of each condition was measured at a 570 nm wavelength in a spectrophotometer (Synergy HT, BioTek, Spain) and normalized to the absorbance from cells cultured on their respective basal media.

### Cardiac Spheroid Generation

hiPSC-CMs and hCFs, separately or mixed at a 70:30 ratio, were suspended in the appropriate media and seeded in U-well-bottom 96-well plates, previously treated with antiadherence rising solution (STEMCELL Technologies, Canada). After 2 days, cells in each well were aggregated into one large spheroid, and aggregates were either maintained in the 96-well plate to track their size or transferred to flask culture bottles in 5–10 mL of new media. The media was changed once a week. For spheroid size monitoring, phase contrast images of at least 10 spheroids per condition were acquired with an optical microscope (Leica THUNDER, Spain), and the diameter was measured using ImageJ open-source software.

### Cell Viability

Cell viability within the spheroids was evaluated by cell membrane integrity analysis. Briefly, spheroids were incubated with 4 μg/mL calcein and 8 μg/mL propidium iodide (PI) in their own media for 24 h at 4 °C. Thereafter, spheroids were protected from light in all steps. Spheroids were rinsed 3 times with PBS and fixed by incubation with 4% PFA at RT and shaking for 40 min. After fixation, spheroids were rinsed again 3 times with PBS and stored in PBS at 4 °C until clearing. The storage period was not prolonged for more than 7 days. For clearing, a modified form^11^ of the CUBIC-2 solution,^12^ hereafter called MD+ clearing solution, was used. MD+ solution was composed of 50% sucrose (w/v), 20% nicotinamide (w/v), 10% triethanolamine (w/v), and 0.1% Triton X-100 (v/v). Spheroids were incubated with 50% MD+ (diluted in distilled water) for 2 h at RT with shaking, followed by incubation with 100% MD+ overnight. Finally, spheroids were visualized under confocal microscopy (Nikon, The Netherlands) and immersed in the same 100% MD+ solution.

To calculate the dead core radius from z-stack viability images, the normalized radial fluorescence intensity of calcein and PI signal was measured with ImageJ at different distance points from the spheroid center. Dead core radius was stablished as the largest distance at which the PI/calcein ratio surpassed a value of 1.

### Histological and Immunofluorescence Analysis of Spheroids

Spheroids were harvested into 1.5 mL tubes, washed with PBS 2 or 3 times, and fixed for 30 min in 4% PFA (VWR, Spain) and 4% sucrose (Sigma-Aldrich, Spain) solution. Fixed spheroids were incubated overnight with 30% sucrose at 4 °C. Then, spheroids were embedded in a Tissue-Tek OCT compound (Sakura) and rapidly frozen by immersion in N2 liquid-cold isopentane (VWR, UK). Blocks of spheroids embedded in the OCT were stored at −80 °C until cryosectioning into layers (thickness of 10 μm) onto SuperFrost Plus glass slides (VWR, Spain).

For immunostaining, samples were permeabilized and blocked by a 30 min incubation at RT in PBS with 0.01% Triton X-100 (Sigma, Spain) and 5% goat serum (Sigma-Aldrich, Germany). Samples were incubated overnight at 4 °C with specific primary antibodies diluted in 0.01% Triton X-100 and 0.5% goat serum in PBS. The primary antibodies used were rabbit antivimentin (diluted 1:500) (IgG polyclonal, SC-7557-R, Santa Cruz Biotechnology), mouse anti-cTnT (diluted 1:200) (13-11, ThermoFisher), rabbit anticaspase-3 (diluted 1:100) (IgG polyclonal, C8487, Sigma), mouse anticollagen-I (COL-1, Santa Cruz Biotechnology), mouse anti collagen-III (B-10, Santa Cruz Biotechnology), and mouse anti- HIF-1-α (diluted 1:50) (28b, Santa Cruz Biotechnology). Next, samples were washed with 0.01% Triton X-100 and 0.5% goat serum in PBS and incubated for 1 h at RT with corresponding secondary antibodies and 0.1 μg/mL Hoechst 33,342 (H1399, Invitrogen) diluted in 0.01% Triton X-100 and 0.5% goat serum in PBS. Secondary antibodies used were goat antimouse AlexaFluor 488 (diluted 1:1000) (A11001, Life Technologies) and goat antirabbit IgG Rhodamine (diluted 1:100) (AP132R, Millipore). Samples were then washed with PBS. Cryosections were covered with DPX mounting solution and cover glasses for storing until fluorescent microscopy visualization (Leica THUNDER, Spain) and further analysis with ImageJ.

For hematoxylin/eosin histological staining, spheroid cryosections were immersed in hematoxylin for 12 min, rinsed with water, immersed in eosin for 20 s, and rinsed with water again. Then, samples were dehydrated by the consecutive incubation in 60° ethanol, 96° ethanol, 100° ethanol (twice), and xylol (twice), for 3 s each. Finally, samples were covered with DPX mounting solution (255,254, ITW Panreac, Spain) and cover glasses for storage until optical microscopy (Leica THUNDER, Spain) inspection.

### Constriction Assay

Spheroid stiffness was determined using a custom-made constriction methacrylate microfluidic device, similar to a previously described system for the microcapsule aspiration assay.^13,14^ The device was designed by BEOnChip S.L. (Spain) and manufactured by Aitiip Centro Tecnológico (Spain). It consists of a single 400 μm tubular channel, which is reduced to 200 μm (the schematic of the device and dimensions are shown in Figure 6). For the constriction assay, the microdevice was connected to a pressure controller (OB1Microfluidic Flow Control System, Elveflow, France) using a 1/16″OD PTFE (Elveflow, France) and TYGON (ACF00002-C, Saint-Gobain, France) tubing systems. The microdevice was placed in an optical inverted microscope (Leica DMi8, Spain) for inspection.

### Constriction Measurements

Spheroids were individually isolated in a U-shaped 96-well plate (Sarstedt, Germany) for optical microscopy inspection (Nikon-Eclipse, the Netherlands). After measuring the spheroid diameter with NIS Elements Analysis Software (Nikon, Netherlands), each spheroid was captured with a micropipet and injected into the system previously perfused with culture media. Pressures lower than 1000 Pa were applied to place the spheroid at the entrance of the microchannel constriction, blocking the passage of the liquid flow. Then, the pressure was increased at a constant rate of 100 Pa/s, pushing the spheroid through. The microscopic optical image of the microdevice and the pressure measurements were video recorded during the whole process for posterior analysis. At least 10 microcapsules and 6 spheroids per condition were tested.

### Stiffness Calculation from Constriction Data

The spheroid stiffness was determined as a pressure-deformation relationship (Δ*P*/Δδ). To obtain this relationship, videos of the constriction assay were processed by using VLC media player software. After each 10 mbar pressure increment, the corresponding video frame was exported as an image file. The length occupied by the deformed spheroid in the 200 μm diameter channel was measured for each exerted pressure (ImageJ software) and normalized by the initial spheroid diameter to calculate deformation (Figure 6). For small deformations, experimental results could be fitted to a linear curve from which the pressure/deformation relationship was obtained, as previously reported for the mechanical characterization of capsules *via* aspiration assays.^14^

### Statistics

Statistical computations were executed by using GraphPad Prism 6 software. Normal distribution was determined by the Shapiro–Wilk normality test. When data followed a normal distribution, one-way ANOVA was used for comparison among multiple groups, followed by Dunnett’s posthoc test. For non-Gaussian data, Kruskal–Wallis’ test was used for comparison among multiple groups, followed by Dunn’s posthoc test. Significance level was set at 0.05.

## Results and Discussion

### Optimization of Cellular Proportions and Culture Medias

Working with cocultures requires careful determination of cellular proportions to properly recreate a human myocardium, along with an adequate medium that satisfies the metabolic needs of both cell types. *In vivo*, CMs represent around 40% of total cell numbers in the human myocardium but occupy almost 75% of the tissue volume due to their large size. On their side, CF numbers are below 20%.^15^ However, these numbers suffer alterations depending on the tissue maturation degree and during disease.^16,17^ Previous attempts to recreate human myocardium *in vitro* have worked with numbers that usually range from 50 to 80% for CM and 15 to 30% for CF, with the inclusion in some cases of other cell types present in the myocardium, such as endothelial cells or mesenchymal stem cells.^9,18−20^ In the absence of a general consensus, we established a tentative 70:30 (hiPSC-CM:hCF) ratio in our models. We chose a high percentage of hiPSC-CM in regard to the small size of our hiPSC-CMs, and considering the fact that hCFs have the potential to grow and proliferate, which is mostly absent in hiPSC-CMs.

**Figure 1:**
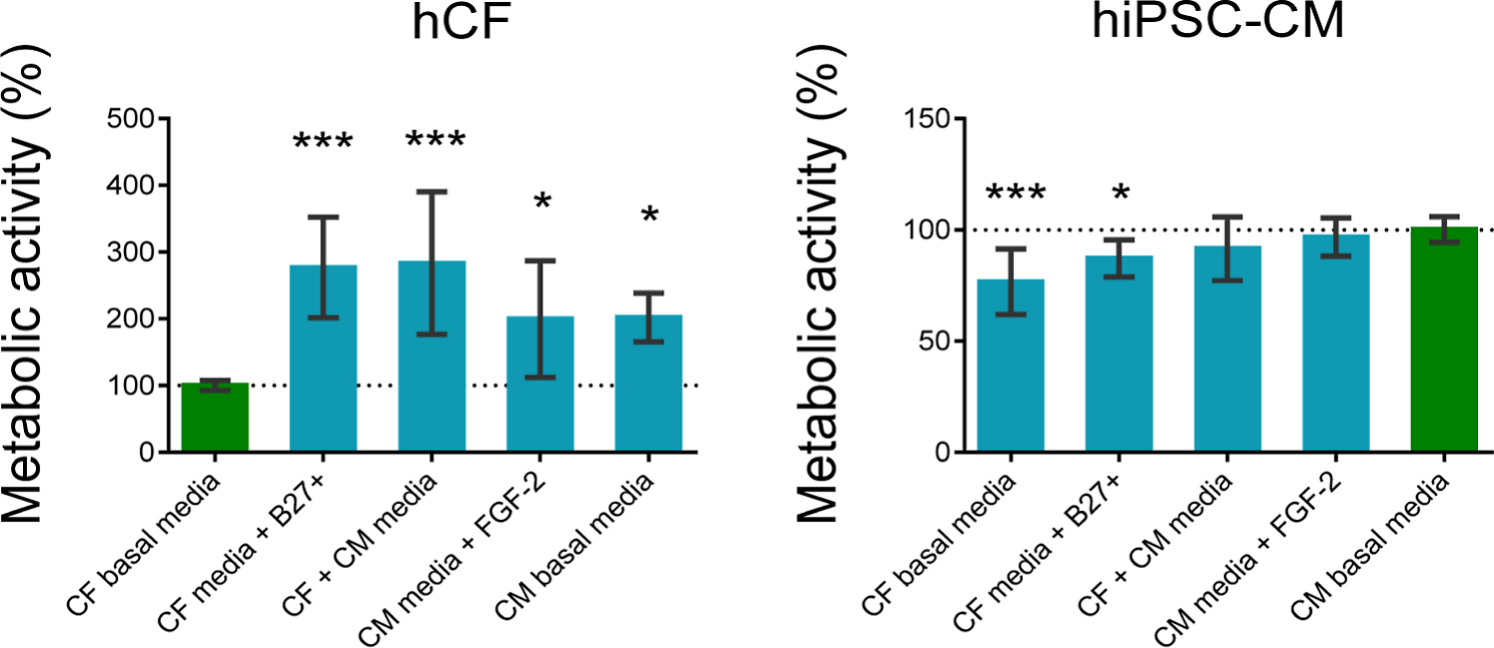
Effect of different media formulations on the metabolic activity of hCF and hiPSC-CM after 7 days of culture. Metabolic activity was measured using the MTT assay. Values represent percentage relative to cell activity in their own basal media (green bars). Bars represent mean ± SD ***: *p* < 0.001 and *: *p* < 0.05 compared to each cell basal media.

Similarly, choosing an adequate media for coculture may be a complex issue. There are many protocols for the monoculture of CMs and CFs that work with different culture media depending on the cell source, among other factors. Regarding the coculture of both cell types, some groups work with specialized commercial media such as iCell or StemPro-34,^21,22^ while others choose a CM basal medium,^20^ since CMs are supposed to be more sensitive to media formulation than fibroblasts. In consensus, the group of D.J. Richards chose a mixture of each cell basal medium in the same ratio than cell proportion in the coculture.^9^ In this context, we have analyzed the effect of different combinations of CM and CF basal media on the metabolic activity of both cell types. hiPSC-CM basal medium consisted of RPMI 1640 supplemented with B27 plus insulin (B27+), while hCF were cultured in low glucose DMEM supplemented with fibroblast growth factor 2 (FGF-2). We studied the effect of both basal media, along with CF basal medium supplemented with B27+, CM basal medium supplemented with FGF-2, and a 1:1 mixture of both basal media (Figure 1).

**Figure 2:**
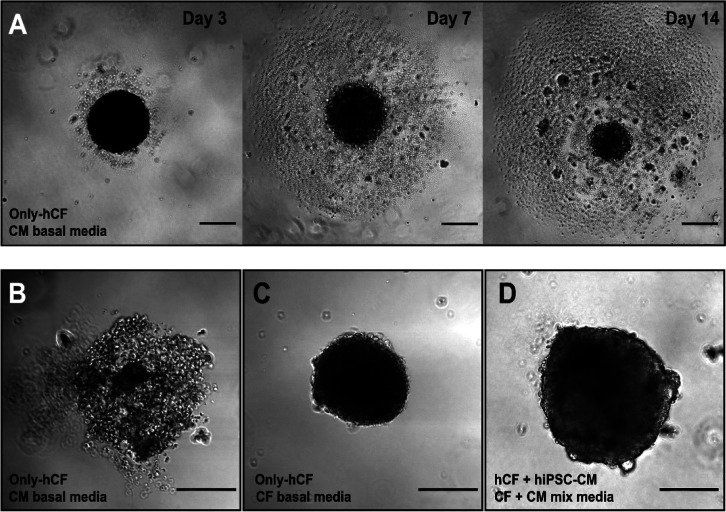
Effect of culture media on cardiac spheroids. (A) Spheroids of 10.000 hCFs cultured in CM basal medium were not compact and cells were lost over time. Detailed images of spheroids after 14 days of culture: spheroid of 10.000 hCFs cultured in (B) CM basal medium and in (C) CF basal medium, and (D) spheroid of 40.000 cells (hiPSC-CM:hCF, 70:30) cultured in CM + CF mix media. Scale bar: 200 μm.

Interestingly, while hiPSC-CMs showed the highest metabolic activity when cultured with CM basal medium, hCF presented the lowest metabolic activity when cultured in their own basal medium, possibly explained by the low concentration of nutrients and growth factors of CF basal medium. In light of these results, we initially chose CM basal medium for the coculture of hiPSC-CM and hCF. However, preliminary attempts to generate single-cell type cardiac spheroids (*i.e.*, only-CM or only-CF spheroids) using CM medium resulted in poor cellular aggregation in only-CF spheroids (Figure 2A,B), in contrast to only-CF spheroids cultured in CF basal medium (Figure 2C). Therefore, a 1:1 mixture of both basal media was finally chosen, ensuring spheroids’ integrity without significant effect on hiPSC-CMs activity compared to CM basal medium. Indeed, hiPSC-CM:hCF spheroids cultured in 1:1 mixture media remained compact for at least 14 days (Figures 2D and S2).

### Generation of Cardiac Spheroids with an Ischemic Core

**Figure 3:**
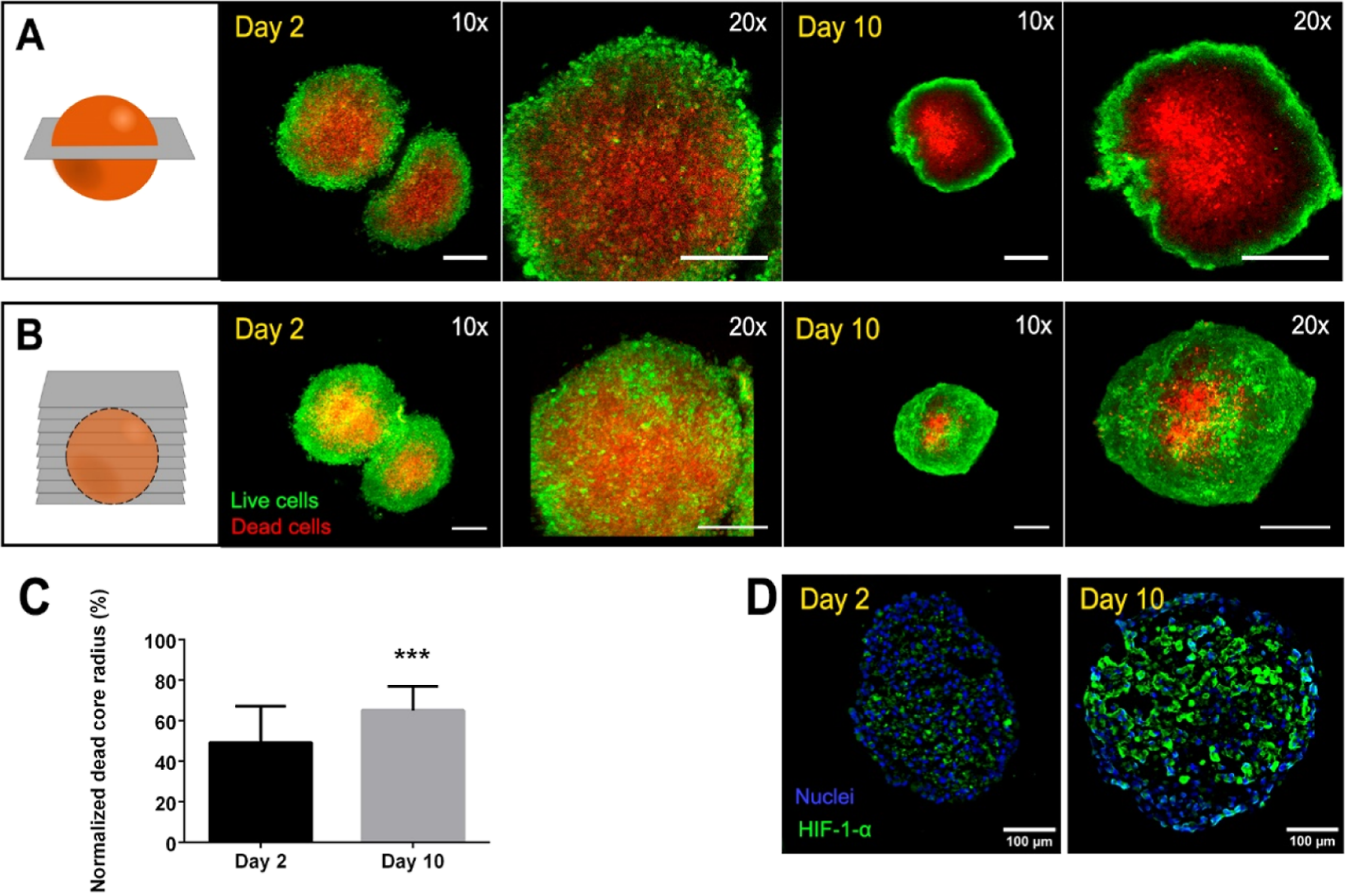
Cellular viability inside cardiac spheroids at day 2 and day 10 of culture. (A) Confocal images of living cells stained with calcein (green) and dead cells stained with propidium iodide (red), followed by spheroid clarification. (B) Top view of the projection from a z-stack of confocal images. Scale bar = 200 μm. (C) Percentage of normalized quantified dead core radius across the multiple spheroids studied for each condition. Bars represent mean ± SD *n* = 7 spheroids with at least 10 z-slides analyzed per spheroid. Mann–Whitney test. ***: *p* < 0.001. (D) HIF-1-α (green) immunostaining images at day 2 and day 10 of culture. Nuclei are depicted in blue.

Once optimal conditions for hiPSC-CM and hCF coculture were established, CM/CF (70:30) spheroids were generated by culture in round-bottom surfaces. Cells were left for 2 days to assemble, transferred to round-bottomed 96-well plates or Petri dishes, and incubated for up to 17 days. To ensure the generation of a hypoxic core, spheroids of at least 500 μm diameter were produced by culturing 40.000 cells per spheroid, considering that the range of oxygen diffusion in spheroids is around 200 μm.^23^ However, the large size of these spheroids constitutes a real handicap for the characterization of ischemic hallmarks. Initially, cellular damage at the ischemic core was assessed by live/dead staining at day 2 and day 10 of culture, but cellular density prevented fluorescent signal visualization a few micrometers below the surface. Thus, after staining, spheroids were fixed and subjected to a clearing process,^11^ which enabled the visualization of inner layers of the spheroid with confocal microscopy (Figure 3A). Z-stack projection of confocal images (Figure 3B) showed the external distribution of living cells and internal congregation of dead cells since day 2 of spheroid culture. Furthermore, at day 10, the frontier between live and dead cells became sharper, with living cells gathering at the spheroid surface and a clearly defined dead core. In fact, quantification of the dead core radius across all the spheroids and conditions showed significant differences (*p* < 0.001) between days 2 and 10, indicating an increased progression of dead cells within the spheroid core (Figure 3C and S3). The dead core evolution overtime correlated with the expression of hypoxia-inducible factor 1-alpha (HIF-1-α), as shown by immunostaining observations (Figure 3D).

Results suggest a quick establishment of ischemia in the defined conditions of spheroid culture (*i.e.*, large spheroids of 40.000 cells, cultured in mixed media without oxygen deprivation). Few works have described the generation of necrotic cores within cardiac spheroids. Thus, deprivation of nutrient and oxygen for 5 h, followed by standard conditions restoration for 16 h, results in apoptosis and cell death after ischemia, exacerbated during reperfusion in only cardiomyocyte spheroids.^6^ More complex spheroids, containing also fibroblasts, subjected to 6 h of nutrient and oxygen reduction closely simulate ischemia,^8^ seeking for a highly hypoxic core and a more functional edge when these cardiac spheroids are incubated for 10 days with a partially reduced oxygen concentration up to 10% O_2_.^9^ However, all of these previously described spheroids require the deprivation of nutrients and other factors, along with the limitation of oxygen to generate ischemia, an external factor overcome in our current model. This supports the representativeness of our system given the speed at which hypoxia affects the natural human myocardium. Additionally, this may reduce experimental times and opens the door to control the timeline of ischemia development by varying the spheroid size.

**Figure 4:**
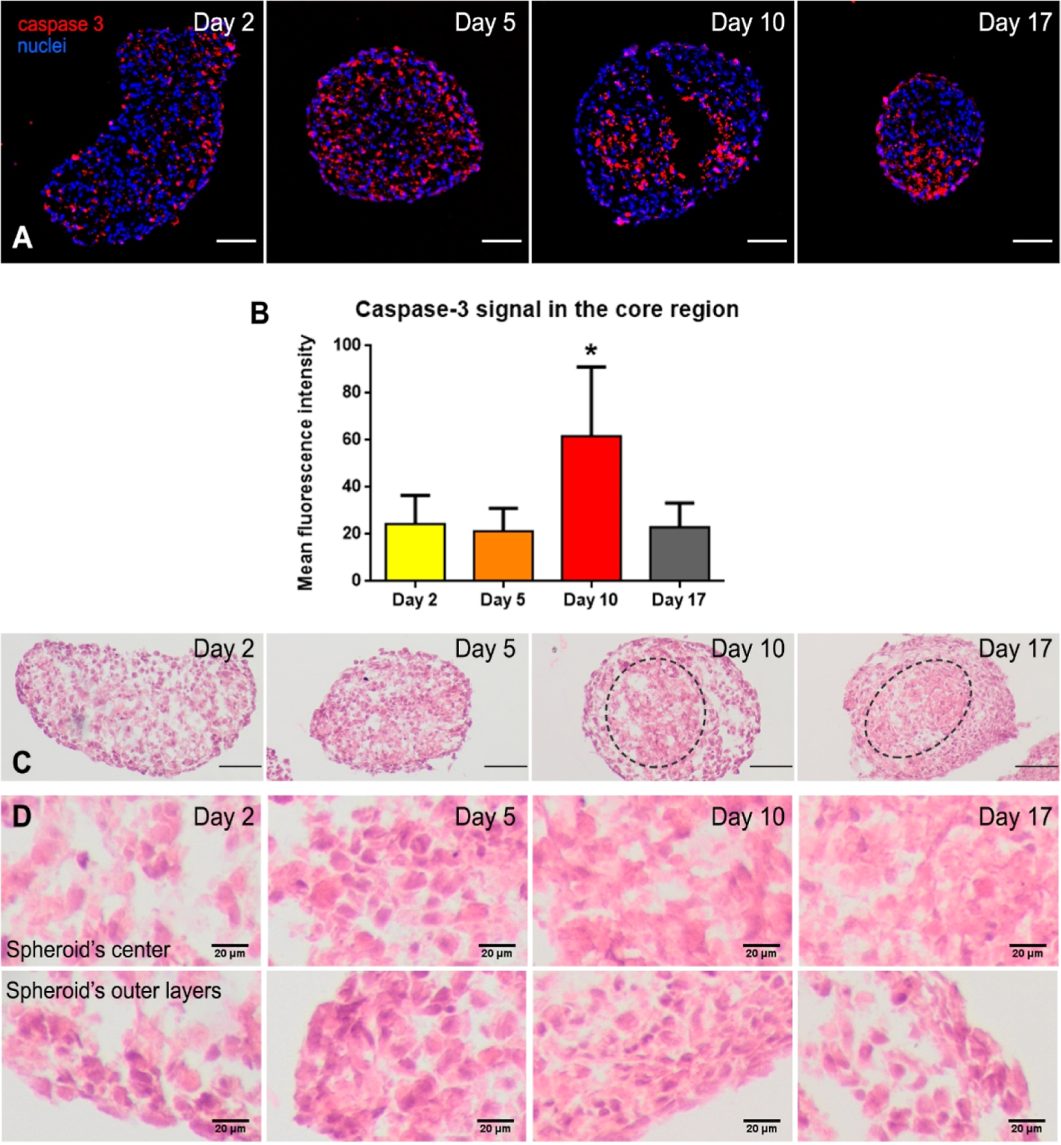
Immunofluorescence and histological analyses of cardiac spheroids. (A) Caspase 3 (apoptosis marker, in red) distribution in cardiac spheroids cryosections. Nuclei were stained with Hoechst (blue). (B) Mean fluorescence intensity of the caspase 3 immunofluorescence signal in the core area (around 10% of the total spheroid area). Bars represent mean ± SD *n* = 17 spheroids. One-way ANOVA with Dunnet’s multiple comparison test. *: *p* < 0.05. (C) Spheroid cryosections stained with hematoxylin/eosin. Delimitation between areas with differential histological features are highlighted with a gray dashed line. Scale bar: 100 μm. (D) Detailed hematoxylin/eosin images of the spheroid center and the outer layers at the defined time points.

Necrosis is classically considered one of the main cell death operators during ischemia/reperfusion injury *in vivo*.^24^ Since there is no direct marker of necrosis, to characterize the cell death pathway inside the spheroids, we investigated the presence of the apoptotic marker caspase-3 by immunofluorescence in spheroid cryosections over time (Figures 4A and S5A). At initial stages of ischemia (days 2 and 5), the spheroid core did not show a high density of apoptotic cells, thus suggesting that necrosis has a leading role. Interestingly, after 10 days of culture, apoptotic cells were concentrated in the inside of the spheroid (Figure 4B), correlating with the live/dead distribution observed at day 10 in whole spheroid confocal images (Figure 3). These findings imply that even if necrosis may initially lead to cell death, at later stages of ischemia, apoptosis may also have a role in the conformation of the ischemic core. In fact, necrosis and apoptosis are not the only death pathways that have been proved to operate during an ischemic event *in vivo*.^25^ Histological images, obtained by hematoxylin/eosin staining of cryosections, confirmed the generation of a death area at day 10 (Figure 4C, gray dot line, and Figure S5B), defined by a characteristic intense pink pigmentation and absence of well delimited nuclei (Figure 4D). Overall, the accumulation of dead cells inside the spheroid supports the recreation of cardiac ischemia by controlling the spheroid size, without alteration of external O_2_.

### Fibroblast Remodeling in Ischemic Cardiac Spheroids

**Figure 5:**
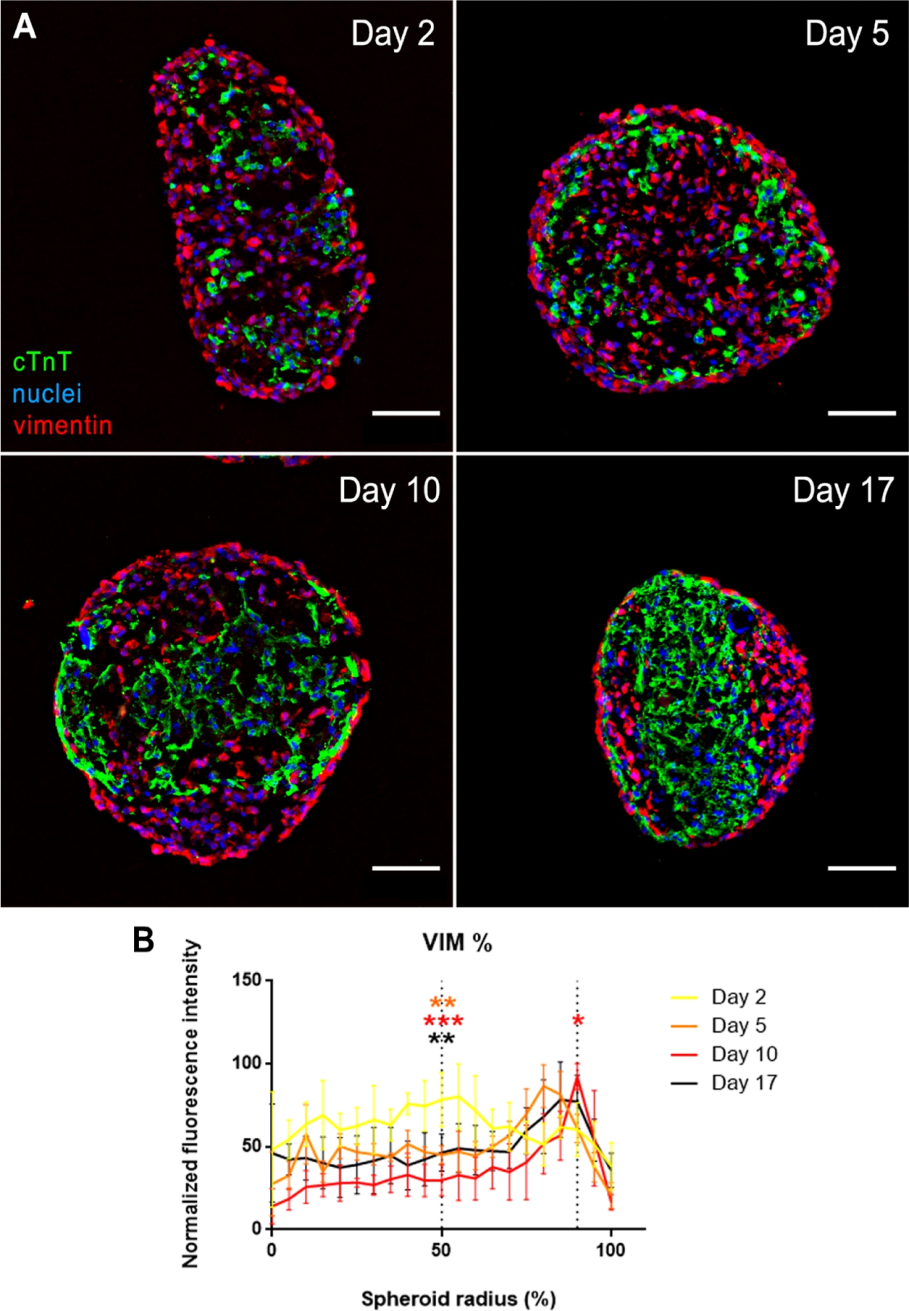
(A) Ischemic-related fibrotic remodeling assessed by immunofluorescence. Vimentin (CF marker, in red) and cTnT (CM marker, in green) disposition across cardiac spheroids cryosections. Nuclei stained with Hoechst (blue). Scale bar = 100 μm. (B) Quantification of spatial distribution of vimentin. Bars represent mean ± SD *n* = 17 spheroids. Statistical significance was assessed at 50 and 90% spheroid radius by one-way ANOVA with Dunnet’s multiple comparison test. *: *p* < 0.05. **: *p* < 0.01 and ***: *p* < 0.001, compared to day 2.

To further corroborate the recreation of ischemia within our large cardiac spheroids, we investigated another consequence of ischemia on the myocardium: the fibrotic tissue remodeling.^7^ In previous models of cardiac ischemia based on the culture of small spheroids on hypoxic conditions,^9^ an increase in vimentin, a CF marker, around the spheroid indicated a fibrotic remodeling that led to spheroid stiffness increase. Thus, we analyzed vimentin and cTnT (CM marker) disposition within spheroids by immunofluorescence staining of spheroid cryosections (Figures 5A and S6). Although in the first days of culture, vimentin and cTnT were randomly arranged within the spheroid, a thin layer of vimentin^+^ cells coating the spheroid surface could already be seen at day 2. After 10 days of culture, the initial disposition evolved to present a central aggrupation of cTnT^+^ cells (*i.e.*, CMs) flanked by areas of vimentin^+^ cells (*i.e.*, CFs), similar to previous observations in smaller cardiac spheroids subjected to external hypoxia, suggesting a shift in fibroblast organization toward the edge of the spheroid.^9^ We confirmed fibroblast redistribution by quantifying the fluorescence signal across the spheroid radius provided by vimentin immunostaining (Figure 5B).

**Figure 6:**
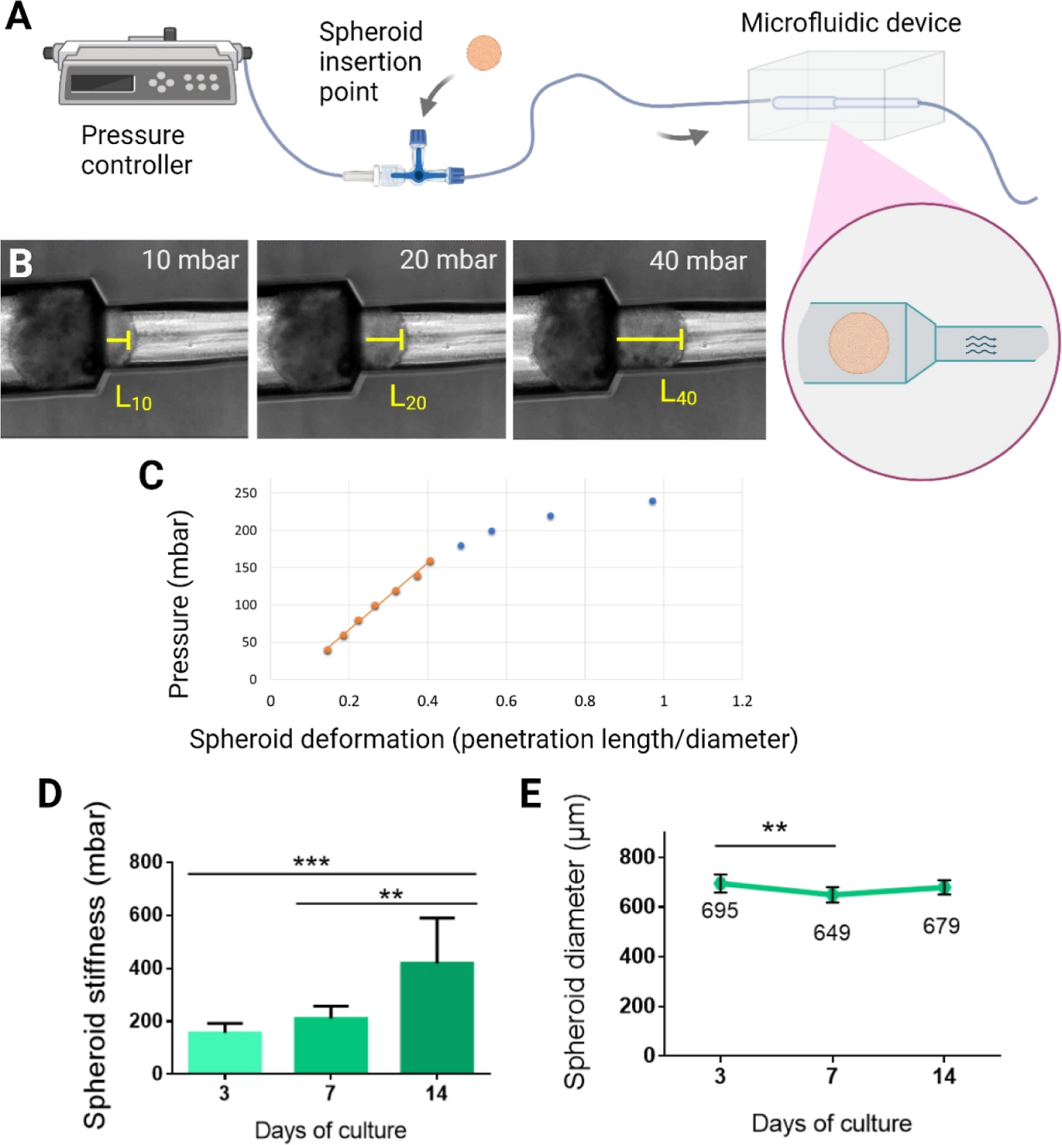
Characterization of the cardiac spheroid stiffness and size through time. (A) Schematic of the constriction assay set up. Spheroids immersed in culture media are injected in a microfluidic device and positioned at the entrance of the microchannel constriction point. By slowly increasing the applied fluidic pressure (B), spheroids are forced to penetrate the narrowest microchannel. For lower pressures, the spheroid deformation (*i.e.*, the penetration length inside the microchannel) and the applied pressure are linearly correlated (C). (D) Spheroid stiffness calculated as the pressure/deformation ratio from a constriction assay. (E) Spheroid radius was measured with Fiji software from bright field images. Bars represent mean ± SD *n* = at least 6 spheroids. One-way ANOVA with Dunnet’s multiple comparison test. ***: *p* < 0.001 and **: *p* < 0.01.

We next investigated if the observed cellular reorganization led to an increase of tissue stiffness, as previously reported.^9^ To study spheroid stiffness, we applied a constriction assay methodology (Figure 6A–C) previously developed in our group for the mechanical characterization of alginate-based microcapsules.^26,27^ Either 20.000 or 40.000 cell seeding spheroids were studied by this system. However, although 20.000 cell seeding spheroids were enough to generate spheroids over 500 μm, they were not retained in the constriction system, not allowing the quantification of the stiffness by this methodology (Figure S4). We found that cardiac spheroids significantly increased their stiffness from day 7 to day 14 of culture (Figure 6D). To discard spheroid compaction as a cause of stiffening, we compared stiffness increment with spheroid size (Figure 6E). Spheroids suffered a slight shrinkage during the first 7 days of culture, while stiffness increment was predominant from day 7 to day 14 of culture, discarding compaction as a cause for stiffness increment, as shown by the relationship between spheroid size and stiffness (Figure S7). Moreover, the timeline of stiffness changes matched the timeline of cellular reorganization, also correlated with a higher collagen type III deposition in the border of the spheroid, without collagen type I expression changes (Figure S8), confirming the initial fibrotic stiffening of cardiac spheroids.^28,29^ Based on these results, and considering that previously described characterization assays are destructive, the extrusion assay under sterile conditions could be used as a nondestructive assay that allows us to track the evolution of the fibrotic stiffening within the spheroid over time.

Overall, our results demonstrate the possibility of recreating cardiac ischemic features by culturing hiPSC-CM and hCF into cardiac spheroids bigger than 500 μm in diameter, with no external stimuli (*i.e.*, nutrient deprivation or hypoxic incubation). Cell death inside the spheroid is already observed at day 2, although high levels of apoptosis and histological features of death appear later, coinciding with a fibrotic remodeling and stiffening of the cardiac spheroid, which are also hints of cardiac ischemia.

## Conclusions

In this work, we assessed the biomimetic recreation of human cardiac ischemia in a 3D *in vitro* model based on self-induction of nutrient, pH, and oxygen gradients that lead to ischemia. We used hiPSC-CMs and hCFs to produce cardiac spheroids. The large size of the spheroids resulted in the generation of a dead core without external inductors of ischemia, such as nutrient deprivation or incubation in hypoxic atmosphere. Cardiac spheroids also recreated hints of ischemic damage pointing to the development of fibrosis, including tissue remodeling and stiffen. Spheroids are easy to culture, do not rely on external matrix that may alter tissue intrinsic features, and are directly accessible for the measure of several parameters, from cellular secretions to beating and response to electric stimulus. Data presented in this work entail the first steps to establish a complex biomimetic spatial recreation of human cardiac ischemia gradients.

## Supporting Information Available

Flow cytometry analysis of cTnT expression in hiPSC-CMs (PDF)

## Abbreviations

CM: cardiomyocyte
cTnT: Cardiac Troponin T
FACS: flow cytometry staining buffer
hADSC: human adipose-derived stem cell
hCF: human cardiac fibroblast
hiPSC: human induced pluripotent stem cell
hiPSC-CM: cardiomyocyte derived from hiPSC
HUVEC: human umbilical vein endothelial cells
IHD: ischemic heart diseases
MTT: 3-[4,5-dimethylthiazol-2-yl]-2,5-diphenyl tetrazolium bromide
PFA: paraformaldehyde

## References

[ref1] Roth G. A.; Mensah G. A.; Johnson C. O.; Addolorato G.; Ammirati E.; Baddour L. M.; Barengo N. C.; Beaton A.; Benjamin E. J.; Benziger C. P.; Bonny A.; Brauer M.; Brodmann M.; Cahill T. J.; Carapetis J. R.; Catapano A. L.; Chugh S.; Cooper L. T.; Coresh J.; Criqui M. H.; DeCleene N. K.; Eagle K. A.; Emmons-Bell S.; Feigin V. L.; Fernández-Sola J.; Fowkes F. G. R.; Gakidou E.; Grundy S. M.; He F. J.; Howard G.; Hu F.; Inker L.; Karthikeyan G.; Kassebaum N. J.; Koroshetz W. J.; Lavie C.; Lloyd-Jones D.; Lu H. S.; Mirijello A.; Misganaw A. T.; Mokdad A. H.; Moran A. E.; Muntner P.; Narula J.; Neal B.; Ntsekhe M.; Oliveira G. M. M.; Otto C. M.; Owolabi M. O.; Pratt M.; Rajagopalan S.; Reitsma M. B.; Ribeiro A. L. P.; Rigotti N. A.; Rodgers A.; Sable C. A.; Shakil S. S.; Sliwa K.; Stark B. A.; Sundström J.; Timpel P.; Tleyjeh I. I.; Valgimigli M.; Vos T.; Whelton P. K.; Yacoub M.; Zuhlke L. J.; Abbasi-Kangevari M.; Abdi A.; Abedi A.; Aboyans V.; Abrha W. A.; Abu-Gharbieh E.; Abushouk A. I.; Acharya D.; Adair T.; Adebayo O. M.; Ademi Z.; Advani S. M.; Afshari K.; Afshin A.; Agarwal G.; Agasthi P.; Ahmad S.; Ahmadi S.; Ahmed M. B.; Aji B.; Akalu Y.; Akande-Sholabi W.; Aklilu A.; Akunna C. J.; Alahdab F.; Al-Eyadhy A.; Alhabib K. F.; Alif S. M.; Alipour V.; Aljunid S. M.; Alla F.; Almasi-Hashiani A.; Almustanyir S.; Al-Raddadi R. M.; Amegah A. K.; Amini S.; Aminorroaya A.; Amu H.; Amugsi D. A.; Ancuceanu R.; Anderlini D.; Andrei T.; Andrei C. L.; Ansari-Moghaddam A.; Anteneh Z. A.; Antonazzo I. C.; Antony B.; Anwer R.; Appiah L. T.; Arabloo J.; Ärnlöv J.; Artanti K. D.; Ataro Z.; Ausloos M.; Avila-Burgos L.; Awan A. T.; Awoke M. A.; Ayele H. T.; Ayza M. A.; Azari S.; Darshan B. B.; Baheiraei N.; Baig A. A.; Bakhtiari A.; Banach M.; Banik P. C.; Baptista E. A.; Barboza M. A.; Barua L.; Basu S.; Bedi N.; Béjot Y.; Bennett D. A.; Bensenor I. M.; Berman A. E.; Bezabih Y. M.; Bhagavathula A. S.; Bhaskar S.; Bhattacharyya K.; Bijani A.; Bikbov B.; Birhanu M. M.; Boloor A.; Brant L. C.; Brenner H.; Briko N. I.; Butt Z. A.; dos Santos F. L. C.; Cahill L. E.; Cahuana-Hurtado L.; Cámera L. A.; Campos-Nonato I. R.; Cantu-Brito C.; Car J.; Carrero J. J.; Carvalho F.; Castañeda-Orjuela C. A.; Catalá-López F.; Cerin E.; Charan J.; Chattu V. K.; Chen S.; Chin K. L.; Choi J. Y. J.; Chu D. T.; Chung S. C.; Cirillo M.; Coffey S.; Conti S.; Costa V. M.; Cundiff D. K.; Dadras O.; Dagnew B.; Dai X.; Damasceno A. A. M.; Dandona L.; Dandona R.; Davletov K.; de la Cruz-Góngora V.; de la Hoz F. P.; de Neve J. W.; Denova-Gutiérrez E.; Molla M. D.; Derseh B. T.; Desai R.; Deuschl G.; Dharmaratne S. D.; Dhimal M.; Dhungana R. R.; Dianatinasab M.; Diaz D.; Djalalinia S.; Dokova K.; Douiri A.; Duncan B. B.; Duraes A. R.; Eagan A. W.; Ebtehaj S.; Eftekhari A.; Eftekharzadeh S.; Ekholuenetale M.; El Nahas N.; Elgendy I. Y.; Elhadi M.; El-Jaafary S. I.; Esteghamati S.; Etisso A. E.; Eyawo O.; Fadhil I.; Faraon E. J. A.; Faris P. S.; Farwati M.; Farzadfar F.; Fernandes E.; Prendes C. F.; Ferrara P.; Filip I.; Fischer F.; Flood D.; Fukumoto T.; Gad M. M.; Gaidhane S.; Ganji M.; Garg J.; Gebre A. K.; Gebregiorgis B. G.; Gebregzabiher K. Z.; Gebremeskel G. G.; Getacher L.; Obsa A. G.; Ghajar A.; Ghashghaee A.; Ghith N.; Giampaoli S.; Gilani S. A.; Gill P. S.; Gillum R. F.; Glushkova E. V.; Gnedovskaya E. V.; Golechha M.; Gonfa K. B.; Goudarzian A. H.; Goulart A. C.; Guadamuz J. S.; Guha A.; Guo Y.; Gupta R.; Hachinski V.; Hafezi-Nejad N.; Haile T. G.; Hamadeh R. R.; Hamidi S.; Hankey G. J.; Hargono A.; Hartono R. K.; Hashemian M.; Hashi A.; Hassan S.; Hassen H. Y.; Havmoeller R. J.; Hay S. I.; Hayat K.; Heidari G.; Herteliu C.; Holla R.; Hosseini M.; Hosseinzadeh M.; Hostiuc M.; Hostiuc S.; Househ M.; Huang J.; Humayun A.; Iavicoli I.; Ibeneme C. U.; Ibitoye S. E.; Ilesanmi O. S.; Ilic I. M.; Ilic M. D.; Iqbal U.; Irvani S. S. N.; Islam S. M. S.; Islam R. M.; Iso H.; Iwagami M.; Jain V.; Javaheri T.; Jayapal S. K.; Jayaram S.; Jayawardena R.; Jeemon P.; Jha R. P.; Jonas J. B.; Jonnagaddala J.; Joukar F.; Jozwiak J. J.; Jürisson M.; Kabir A.; Kahlon T.; Kalani R.; Kalhor R.; Kamath A.; Kamel I.; Kandel H.; Kandel A.; Karch A.; Kasa A. S.; Katoto P. D. M. C.; Kayode G. A.; Khader Y. S.; Khammarnia M.; Khan M. M. S. M. N.; Khan M. M. S. M. N.; Khan M. M. S. M. N.; Khan E. A.; Khatab K.; Kibria G. M. A.; Kim Y. J.; Kim G. R.; Kimokoti R. W.; Kisa S.; Kisa A.; Kivimäki M.; Kolte D.; Koolivand A.; Korshunov V. A.; Laxminarayana S. L. K.; Koyanagi A.; Krishan K.; Krishnamoorthy V.; Defo B. K.; Bicer B. K.; Kulkarni V.; Kumar G. A.; Kumar N.; Kurmi O. P.; Kusuma D.; Kwan G. F.; la Vecchia C.; Lacey B.; Lallukka T.; Lan Q.; Lasrado S.; Lassi Z. S.; Lauriola P.; Lawrence W. R.; Laxmaiah A.; LeGrand K. E.; Li M. C.; Li B.; Li S.; Lim S. S.; Lim L. L.; Lin H.; Lin Z.; Lin R. T.; Liu X.; Lopez A. D.; Lorkowski S.; Lotufo P. A.; Lugo A.; Nirmal K. M.; Madotto F.; Mahmoudi M.; Majeed A.; Malekzadeh R.; Malik A. A.; Mamun A. A.; Manafi N.; Mansournia M. A.; Mantovani L. G.; Martini S.; Mathur M. R.; Mazzaglia G.; Mehata S.; Mehndiratta M. M.; Meier T.; Menezes R. G.; Meretoja A.; Mestrovic T.; Miazgowski B.; Miazgowski T.; Michalek I. M.; Miller T. R.; Mirrakhimov E. M.; Mirzaei H.; Moazen B.; Moghadaszadeh M.; Mohammad Y.; Mohammad D. K.; Mohammed S.; Mohammed M. A.; Mokhayeri Y.; Molokhia M.; Montasir A. A.; Moradi G.; Moradzadeh R.; Moraga P.; Morawska L.; Velásquez I. M.; Morze J.; Mubarik S.; Muruet W.; Musa K. I.; Nagarajan A. J.; Nalini M.; Nangia V.; Naqvi A. A.; Swamy S. N.; Nascimento B. R.; Nayak V. C.; Nazari J.; Nazarzadeh M.; Negoi R. I.; Kandel S. N.; Nguyen H. L. T.; Nixon M. R.; Norrving B.; Noubiap J. J.; Nouthe B. E.; Nowak C.; Odukoya O. O.; Ogbo F. A.; Olagunju A. T.; Orru H.; Ortiz A.; Ostroff S. M.; Padubidri J. R.; Palladino R.; Pana A.; Panda-Jonas S.; Parekh U.; Park E. C.; Parvizi M.; Kan F. P.; Patel U. K.; Pathak M.; Paudel R.; Pepito V. C. F.; Perianayagam A.; Perico N.; Pham H. Q.; Pilgrim T.; Piradov M. A.; Pishgar F.; Podder V.; Polibin R. V.; Pourshams A.; Pribadi D. R. A.; Rabiee N.; Rabiee M.; Radfar A.; Rafiei A.; Rahim F.; Rahimi-Movaghar V.; Rahman M. A. M. H. U.; Rahman M. A. M. H. U.; Rahmani A. M.; Rakovac I.; Ram P.; Ramalingam S.; Rana J.; Ranasinghe P.; Rao S. J.; Rathi P.; Rawal L.; Rawasia W. F.; Rawassizadeh R.; Remuzzi G.; Renzaho A. M. N.; Rezapour A.; Riahi S. M.; Roberts-Thomson R. L.; Roever L.; Rohloff P.; Romoli M.; Roshandel G.; Rwegerera G. M.; Saadatagah S.; Saber-Ayad M. M.; Sabour S.; Sacco S.; Sadeghi M.; Moghaddam S. S.; Safari S.; Sahebkar A.; Salehi S.; Salimzadeh H.; Samaei M.; Samy A. M.; Santos I. S.; Santric-Milicevic M. M.; Sarrafzadegan N.; Sarveazad A.; Sathish T.; Sawhney M.; Saylan M.; Schmidt M. I.; Schutte A. E.; Senthilkumaran S.; Sepanlou S. G.; Sha F.; Shahabi S.; Shahid I.; Shaikh M. A.; Shamali M.; Shamsizadeh M.; Shawon M. S. R.; Sheikh A.; Shigematsu M.; Shin M. J.; Shin J. I.; Shiri R.; Shiue I.; Shuval K.; Siabani S.; Siddiqi T. J.; Silva D. A. S.; Singh J. A.; Singh A.; Skryabin V. Y.; Skryabina A. A.; Soheili A.; Spurlock E. E.; Stockfelt L.; Stortecky S.; Stranges S.; Abdulkader R. S.; Tadbiri H.; Tadesse E. G.; Tadesse D. B.; Tajdini M.; Tariqujjaman M.; Teklehaimanot B. F.; Temsah M. H.; Tesema A. K.; Thakur B.; Thankappan K. R.; Thapar R.; Thrift A. G.; Timalsina B.; Tonelli M.; Touvier M.; Tovani-Palone M. R.; Tripathi A.; Tripathy J. P.; Truelsen T. C.; Tsegay G. M.; Tsegaye G. W.; Tsilimparis N.; Tusa B. S.; Tyrovolas S.; Umapathi K. K.; Unim B.; Unnikrishnan B.; Usman M. S.; Vaduganathan M.; Valdez P. R.; Vasankari T. J.; Velazquez D. Z.; Venketasubramanian N.; Vu G. T.; Vujcic I. S.; Waheed Y.; Wang Y.; Wang F.; Wei J.; Weintraub R. G.; Weldemariam A. H.; Westerman R.; Winkler A. S.; Wiysonge C. S.; Wolfe C. D. A.; Wubishet B. L.; Xu G.; Yadollahpour A.; Yamagishi K.; Yan L. L.; Yandrapalli S.; Yano Y.; Yatsuya H.; Yeheyis T. Y.; Yeshaw Y.; Yilgwan C. S.; Yonemoto N.; Yu C.; Yusefzadeh H.; Zachariah G.; Zaman S. B.; Zaman M. S.; Zamanian M.; Zand R.; Zandifar A.; Zarghi A.; Zastrozhin M. S.; Zastrozhina A.; Zhang Z. J.; Zhang Y.; Zhang W.; Zhong C.; Zou Z.; Zuniga Y. M. H.; Murray C. J. L.; Fuster V. Global Burden of Cardiovascular Diseases and Risk Factors, 1990–2019: Update From the GBD 2019 Study. J. Am. Coll. Cardiol. 2020, 76 (25), 2982–3021. 10.1016/J.JACC.2020.11.010/SUPPL_FILE/MMC3.DOCX. 33309175 PMC7755038

[ref2] Zuppinger C. 3D Cardiac Cell Culture: A Critical Review of Current Technologies and Applications. Front. Cardiovasc. Med. 2019, 6, 87 10.3389/fcvm.2019.00087. 31294032 PMC6606697

[ref3] Soon K.; Mourad O.; Nunes S. S. Engineered Human Cardiac Microtissues: The State-of-the-(He)Art. Stem Cell. 2021, 39 (8), 1008–1016. 10.1002/stem.3376. 33786918

[ref4] Paz-Artigas L.; Montero-Calle P.; Iglesias-García O.; Mazo M. M.; Ochoa I.; Ciriza J. Current Approaches for the Recreation of Cardiac Ischaemic Environment in Vitro. Int. J. Pharm. 2023, 632, 122589 10.1016/j.ijpharm.2023.122589. 36623742

[ref5] Steenbergen C.; Frangogiannis N. G. Ischemic Heart Disease. In Muscle: Fundamental Biology and Mechanisms of Disease; Hill J. A., Olson E. N., Eds.; Elsevier Inc., 2012; Vol. 1, pp 495–521.

[ref6] Sebastião M. J.; Gomes-Alves P.; Reis I.; Sanchez B.; Palacios I.; Serra M.; Alves P. M. Bioreactor-Based 3D Human Myocardial Ischemia/Reperfusion in Vitro Model: A Novel Tool to Unveil Key Paracrine Factors upon Acute Myocardial Infarction. Transl. Res. 2020, 215, 57–74. 10.1016/j.trsl.2019.09.001. 31541616

[ref7] Mazo M.; Pelacho B.; Prósper F. Stem Cell Therapy for Chronic Myocardial Infarction. J. Cardiovasc. Transl. Res. 2010, 3 (2), 79–88. 10.1007/s12265-009-9159-9. 20560022

[ref8] Richards D. J.; Coyle R. C.; Tan Y.; Jia J.; Wong K.; Toomer K.; Menick D. R.; Mei Y. Inspiration from Heart Development: Biomimetic Development of Functional Human Cardiac Organoids. Biomaterials 2017, 142, 112–123. 10.1016/j.biomaterials.2017.07.021. 28732246 PMC5562398

[ref9] Richards D. J.; Li Y.; Kerr C. M.; Yao J.; Beeson G. C.; Coyle R. C.; Chen X.; Jia J.; Damon B.; Wilson R.; Starr Hazard E.; Hardiman G.; Menick D. R.; Beeson C. C.; Yao H.; Ye T.; Mei Y.; Starr Hazard E.; Hardiman G.; Menick D. R.; Beeson C. C.; Yao H.; Ye T.; Mei Y.; Hazard E. S.; Hardiman G.; Menick D. R.; Beeson C. C.; Yao H.; Ye T.; Mei Y. Human Cardiac Organoids for the Modelling of Myocardial Infarction and Drug Cardiotoxicity. Nat. Biomed. Eng. 2020, 4 (4), 446–462. 10.1038/s41551-020-0539-4. 32284552 PMC7422941

[ref10] Burridge P. W.; Matsa E.; Shukla P.; Lin Z. C.; Churko J. M.; Ebert A. D.; Lan F.; Diecke S.; Huber B.; Mordwinkin N. M.; Plews J. R.; Abilez O. J.; Cui B.; Gold J. D.; Wu J. C. Chemically Defined Generation of Human Cardiomyocytes. Nat. Methods 2014, 11 (8), 855–860. 10.1038/nmeth.2999. 24930130 PMC4169698

[ref11] Lempereur S.; Machado E.; Licata F.; Buzer L.; Robineau I.; Hémon J.; Banerjee P.; De Crozé N.; Léonard M.; Affaticati P.; Jenett A.; Talbot H.; Joly J.-S. ZeBraInspector, a Whole Organism Screening Platform Enabling Volumetric Analysis of Zebrafish Brain White Matter. bioRxiv 2020, 10.1101/2020.10.26.353656.

[ref12] Susaki E. A.; Tainaka K.; Perrin D.; Yukinaga H.; Kuno A.; Ueda H. R. Advanced CUBIC Protocols for Whole-Brain and Whole-Body Clearing and Imaging. Nat. Protoc. 2015, 10 (11), 1709–1727. 10.1038/nprot.2015.085. 26448360

[ref13] Kleinberger R. M.; Burke N. A. D.; Dalnoki-Veress K.; Stöver H. D. Systematic Study of Alginate-Based Microcapsules by Micropipette Aspiration and Confocal Fluorescence Microscopy. Mater. Sci. Eng., C 2013, 33 (7), 4295–4304. 10.1016/j.msec.2013.06.033. 23910346

[ref14] Le Goff A.; Kaoui B.; Kurzawa G.; Haszon B.; Salsac A. V. Squeezing Bio-Capsules into a Constriction: Deformation till Break-Up. Soft Matter 2017, 13 (41), 7644–7648. 10.1039/C7SM01417A. 28990040

[ref15] Litviňuková M.; Talavera-López C.; Maatz H.; Reichart D.; Worth C. L.; Lindberg E. L.; Kanda M.; Polanski K.; Heinig M.; Lee M.; Nadelmann E. R.; Roberts K.; Tuck L.; Fasouli E. S.; DeLaughter D. M.; McDonough B.; Wakimoto H.; Gorham J. M.; Samari S.; Mahbubani K. T.; Saeb-Parsy K.; Patone G.; Boyle J. J.; Zhang H.; Zhang H.; Viveiros A.; Oudit G. Y.; Bayraktar O. A.; Seidman J. G.; Seidman C. E.; Noseda M.; Hubner N.; Teichmann S. A. Cells of the Adult Human Heart. Nature 2020, 588 (7838), 466–472. 10.1038/s41586-020-2797-4. 32971526 PMC7681775

[ref16] Bednarowicz K. A.; Kurpisz M. Biological Bases of Cardiac Function and the Pro-Regenerative Potential of Stem Cells in the Treatment of Myocardial Disorder. Cardiac Cell Culture Technologies: Microfluidic and On-Chip Systems; Springer: Cham, 2017; pp 79–108.

[ref17] Zhang P.; Su J.; Mende U. Cross Talk between Cardiac Myocytes and Fibroblasts: From Multiscale Investigative Approaches to Mechanisms and Functional Consequences. Am. J. Physiol.: Heart Circ. Physiol. 2012, 303 (12), H1385–H1396. 10.1152/ajpheart.01167.2011. 23064834 PMC3532535

[ref18] Yang B.; Lui C.; Yeung E.; Matsushita H.; Jeyaram A.; Pitaktong I.; Inoue T.; Mohamed Z.; Ong C. S.; Disilvestre D.; Jay S. M.; Tung L.; Tomaselli G.; Ma C.; Hibino N.; Yang B.; Lui C.; Yeung E.; Matsushita H.; Jeyaram A.; Pitaktong I.; Inoue T.; Mohamed Z.; Siang O.; DiSilvestre D.; M J.; Tung L.; Tomaselli G.; Ma C.; Hibino N.; Yang B.; Lui C.; Yeung E.; Matsushita H.; Jeyaram A.; Pitaktong I.; Inoue T.; Mohamed Z.; Ong C. S.; Disilvestre D.; Jay S. M.; Tung L.; Tomaselli G.; Ma C.; Hibino N. A Net Mold-Based Method of Biomaterial-Free Three-Dimensional Cardiac Tissue Creation. Tissue Eng., Part C 2019, 25 (4), 243–252. 10.1089/ten.tec.2019.0003. PMC13215059 30913987

[ref19] Polonchuk L.; Chabria M.; Badi L.; Hoflack J.-C. C.; Figtree G.; Davies M. J.; Gentile C. Cardiac Spheroids as Promising in Vitro Models to Study the Human Heart Microenvironment. Sci. Rep. 2017, 7 (1), 7005–7012. 10.1038/s41598-017-06385-8. 28765558 PMC5539326

[ref20] Veldhuizen J.; Chavan R.; Moghadas B.; Park J. G.; Kodibagkar V. D.; Migrino R. Q.; Nikkhah M. Cardiac Ischemia On-a-Chip to Investigate Cellular and Molecular Response of Myocardial Tissue under Hypoxia. Biomaterials 2022, 281, 121336 10.1016/j.biomaterials.2021.121336. 35026670 PMC10440189

[ref21] Mastikhina O.; Moon B. U.; Williams K.; Hatkar R.; Gustafson D.; Mourad O.; Sun X.; Koo M.; Lam A. Y. L.; Sun Y.; Fish J. E.; Young E. W. K.; Nunes S. S. Human Cardiac Fibrosis-on-a-Chip Model Recapitulates Disease Hallmarks and Can Serve as a Platform for Drug Testing. Biomaterials 2020, 233, 119741 10.1016/j.biomaterials.2019.119741. 31927251

[ref22] Daly A. C.; Davidson M. D.; Burdick J. A.; Hatkar R.; Gustafson D.; Mourad O.; Sun X.; Koo M.; Lam A. Y. L.; Sun Y.; Fish J. E.; Young E. W. K.; Nunes S. S.; Daly A. C.; Davidson M. D.; Burdick J. A. 3D Bioprinting of High Cell-Density Heterogeneous Tissue Models through Spheroid Fusion within Self-Healing Hydrogels. Nat. Commun. 2021, 12 (1), 753–813. 10.1038/s41467-021-21029-2. 33531489 PMC7854667

[ref23] Carlsson J.; Acker H. Relations between PH, Oxygen Partial Pressure and Growth in Cultured Cell Spheroids. Int. J. Cancer 1988, 42 (5), 715–720. 10.1002/ijc.2910420515. 3182108

[ref24] Del Re D. P.; Amgalan D.; Linkermann A.; Liu Q.; Kitsis R. N. Fundamental Mechanisms of Regulated Cell Death and Implications for Heart Disease. Physiol. Rev. 2019, 99 (4), 1765–1817. 10.1152/physrev.00022.2018. 31364924 PMC6890986

[ref25] Davidson S. M.; Adameová A.; Barile L.; Cabrera-Fuentes H. A.; Lazou A.; Pagliaro P.; Stensløkken K.; Garcia-Dorado D. Mitochondrial and Mitochondrial-Independent Pathways of Myocardial Cell Death during Ischaemia and Reperfusion Injury. J. Cell. Mol. Med. 2020, 24 (7), 3795–3806. 10.1111/jcmm.15127. 32155321 PMC7171390

[ref26] Virumbrales-Muñoz M.; Paz-Artigas L.; Ciriza J.; Alcaine C.; Espona-Noguera A.; Doblaré M.; Sáenz del Burgo L.; Ziani K.; Pedraz J. L.; Fernández L.; Ochoa I. Force Spectroscopy Imaging and Constriction Assays Reveal the Effects of Graphene Oxide on the Mechanical Properties of Alginate Microcapsules. ACS Biomater. Sci. Eng. 2021, 7 (1), 242–253. 10.1021/acsbiomaterials.0c01382. 33337130

[ref27] Paz-Artigas L.; Ziani K.; Alcaine C.; Báez-Díaz C.; Blanco-Blázquez V.; Pedraz J. L.; Ochoa I.; Ciriza J. Benefits of Cryopreservation as Long-Term Storage Method of Encapsulated Cardiosphere-Derived Cells for Cardiac Therapy: A Biomechanical Analysis. Int. J. Pharm. 2021, 607, 121014 10.1016/j.ijpharm.2021.121014. 34400275

[ref28] Inoue K.; Kusachi S.; Niiya K.; Kajikawa Y.; Tsuji T. Sequential Changes in the Distribution of Type I and III Collagens in the Infarct Zone: Immunohistochemical Study of Experimental Myocardial Infarction in the Rat. Coron. Artery Dis. 1995, 6 (2), 153–158. 10.1097/00019501-199502000-00010. 7780621

[ref29] Cleutjens J. P. M.; Verluyten M. J. A.; Smits J. F. M.; Daemen M. J. A. P. Collagen Remodeling after Myocardial Infarction in the Rat Heart. Am. J. Pathol. 1995, 147 (2), 325–338. 7639329 PMC1869816

