# Supplementary material for: Generation of Self-Induced Myocardial Ischemia in Large-Sized Cardiac Spheroids without Alteration of Environmental Conditions Recreates Fibrotic Remodeling and Tissue Stiffening Revealed by Constriction Assays
Source: ACS Biomater Sci Eng. 2024 Jan 18;10(2):987–97. doi: 10.1021/acsbiomaterials.3c01302 (PMC10865285; doi:10.1021/acsbiomaterials.3c01302)
Supplement: Supplementary file 1 — ab3c01302_si_001.pdf [file ab3c01302_si_001.pdf]

Generation of self-induced myocardial ischemia in large size cardiac spheroids without alteration of environmental conditions recreates fibrotic remodelling and tissue stiffening revealed by constriction assay

Laura Paz-Artigas[a,b], Sandra González-Lana[a,c], Nicolás Polo[a], Pedro Vicente[d,e], Pilar Montero-Calle[g], Miguel A. Martínez[a,f], Gregorio Rábago[g], Margarida Serra[d,e], Felipe Prósper[h,i,j], Manuel M. Mazo[h,i], Arantxa González[k,l], Ignacio Ochoa[a,b,f], Jesús Ciriza[a,b,f]**

[a] Tissue Microenvironment (TME) Lab, Aragón Institute of Engineering Research (I3A), University of Zaragoza, 50018 Zaragoza, Spain

[b] Institute for Health Research Aragón (IIS Aragón), 50009 Zaragoza, Spain

[c] BEONCHIP S.L., CEMINEM, Campus Río Ebro, 50018 Zaragoza, Spain

[d] Instituto de Biologia Experimental e Tecnológica (iBET), 2780-157 Oeiras, Portugal

[e] Instituto de Tecnologia Química e Biológica António Xavier, Universidade Nova de Lisboa, 2780-157 Oeiras, Portugal

[f] CIBER-BBN, ISCIII, 50018 Zaragoza, Spain

[g] Cardiology and Cardiac Surgery Department, Clínica Universidad de Navarra, 31009 Pamplona, Spain

[h] Regenerative Medicine Program, Cima Universidad de Navarra, and Instituto de Investigación Sanitaria de Navarra (IdiSNA), 31008 Pamplona, Spain

[i] Hematology and Cell Therapy, Clínica Universidad de Navarra, and Instituto de Investigación Sanitaria de Navarra (IdiSNA), 31008 Pamplona, Spain

[j] CIBERONC, Instituto de Salud Carlos III, 28029 Madrid, Spain

[k] Program of Cardiovascular Diseases, CIMA Universidad de Navarra, and Instituto de Investigación Sanitaria de Navarra (IdiSNA), 31008 Pamplona, Spain

[l] CIBERCV, Instituto de Salud Carlos III, 28029 Madrid, Spain

*Corresponding author: Jesús Ciriza, PhD; Ignacio Ochoa, PhD

University of Zaragoza - I3A | IIS Aragón || CIBER-BBN

Campus Rio Ebro, Mariano Esquillor s/n, Edificio I+D+I, 50018 Zaragoza

Phone: +34 876 55 5157

E-mail address: jeciriza@unizar.es, iochgar@unizar.es

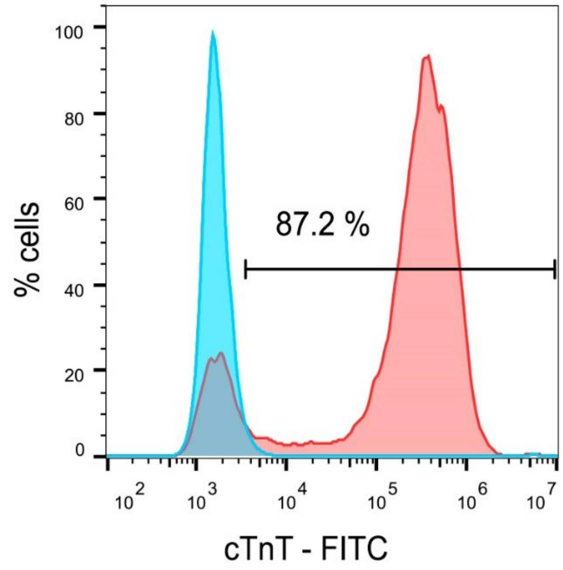

Figure S1. Flow cytometry analysis of cardiac Troponin T (cTnT) expression in hiPSC-CMs.

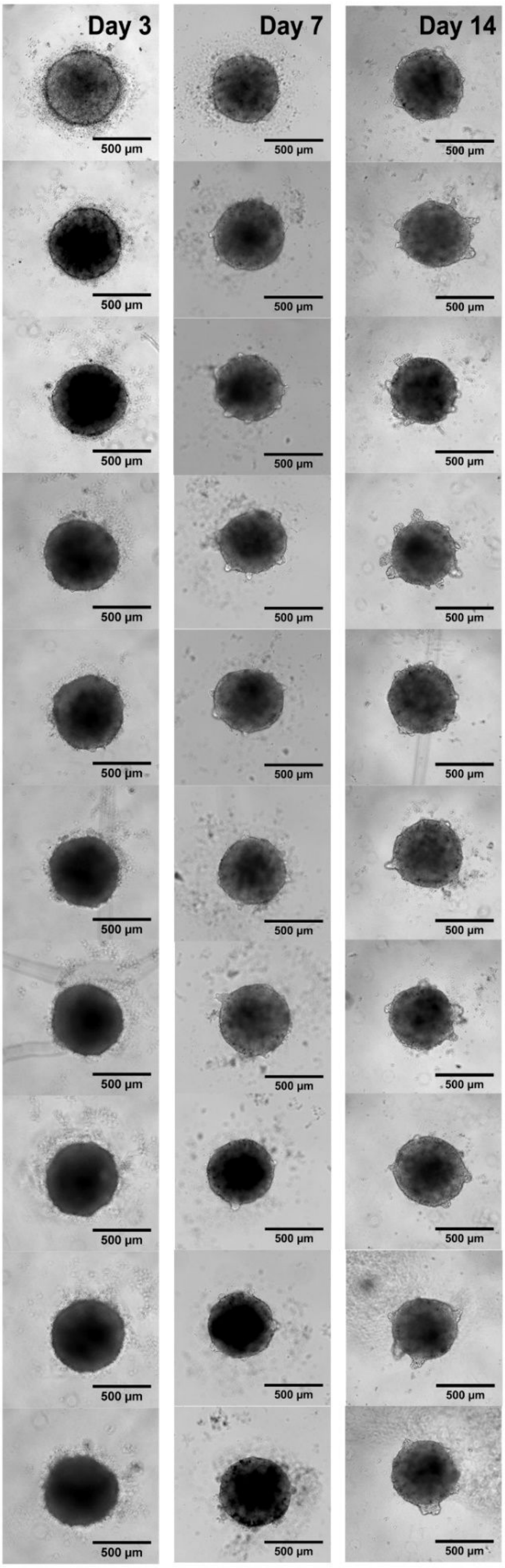

Figure S2. Spheroids of 40.000 cells (hiPSC-CM:hCF, 70:30) cultured in CM+CF mix media for 14 days.

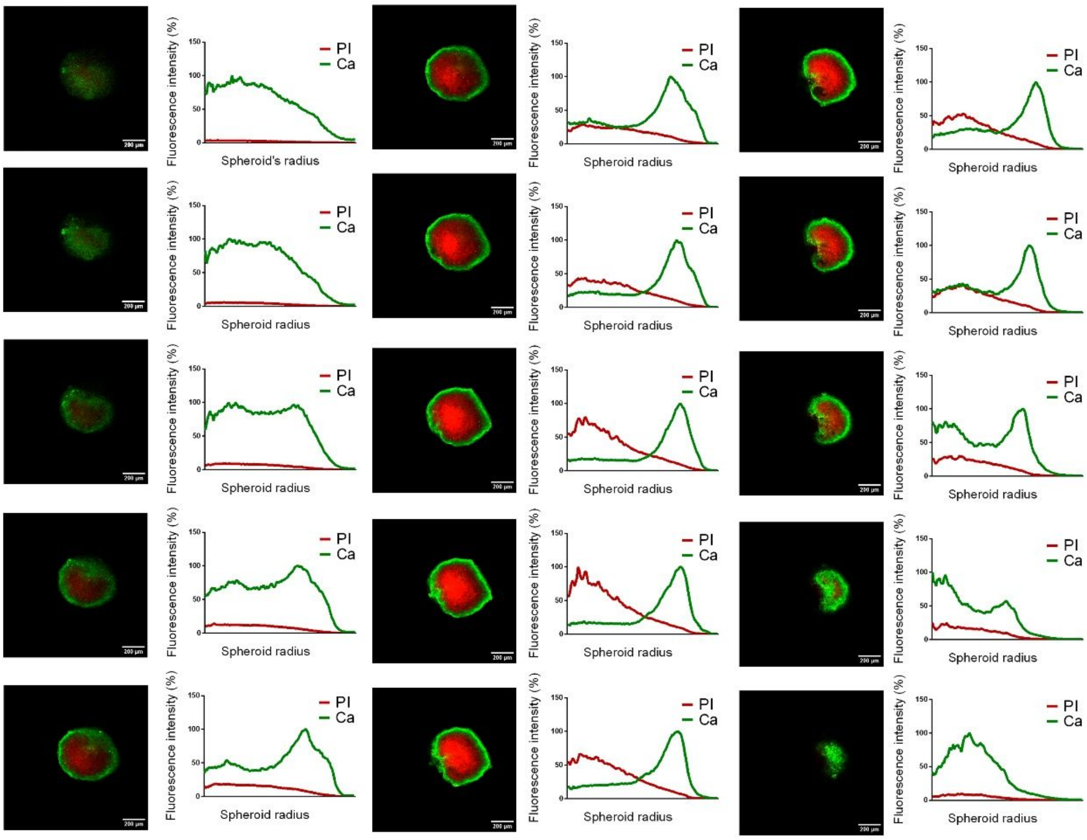

Figure S3. Images and fluorescence quantifications from the Z slices from one spheroid (red line: propidium iodide, green line: calcein).

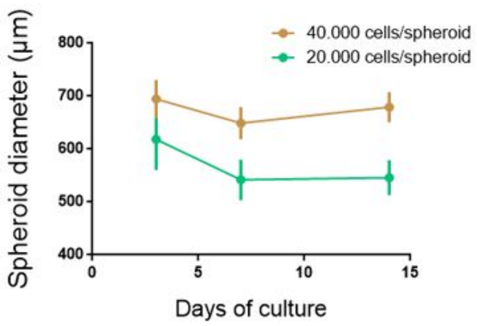

Figure S4. Diameter of spheroids overtime

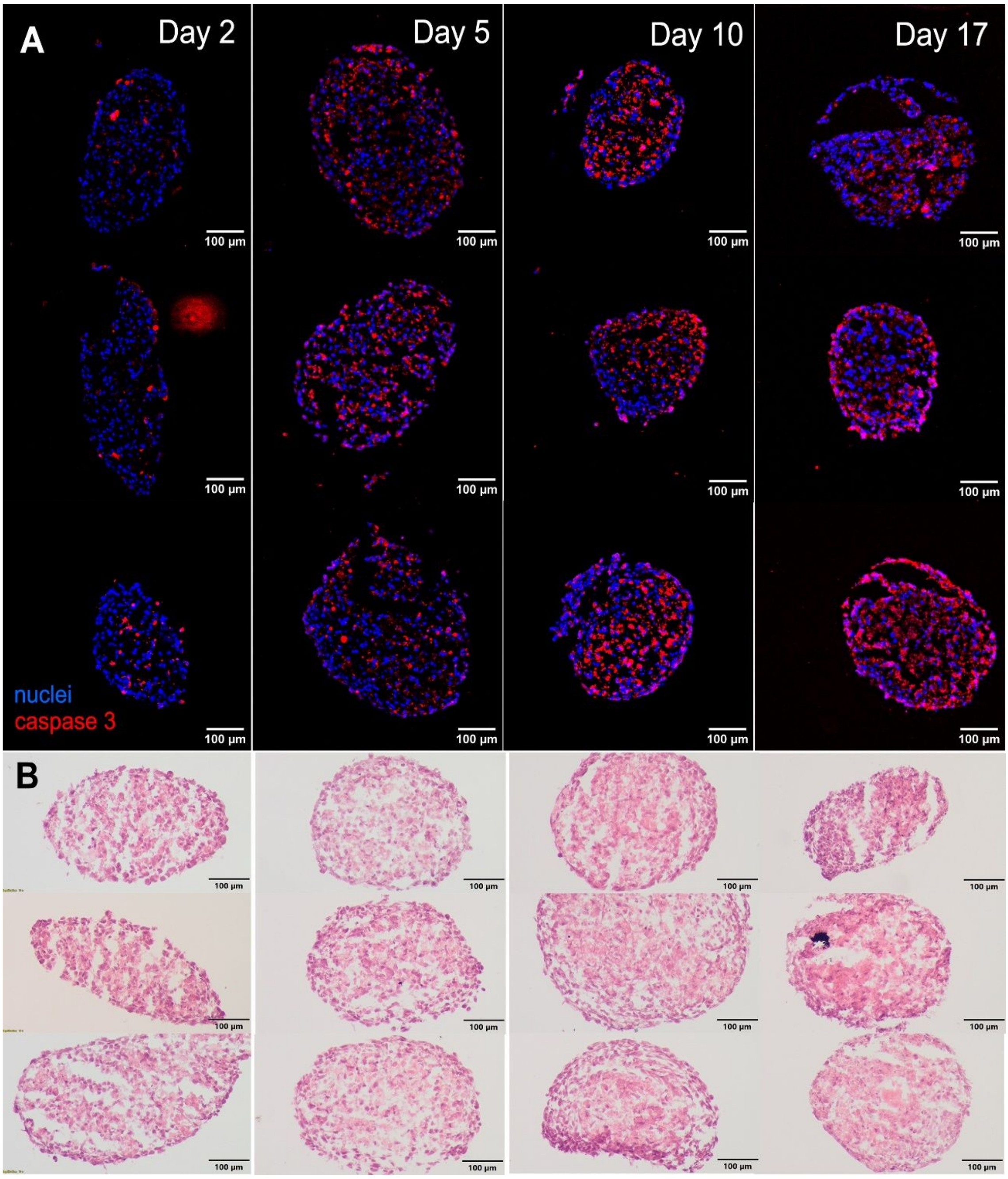

Figure S5. A) Caspase 3 (apoptosis marker, in red) distribution in cardiac spheroids cryosections at defined timepoints. Nuclei stained with Hoechst (blue). B) Spheroid cryosections stained with haematoxylin/eosin at the defined timepoints.

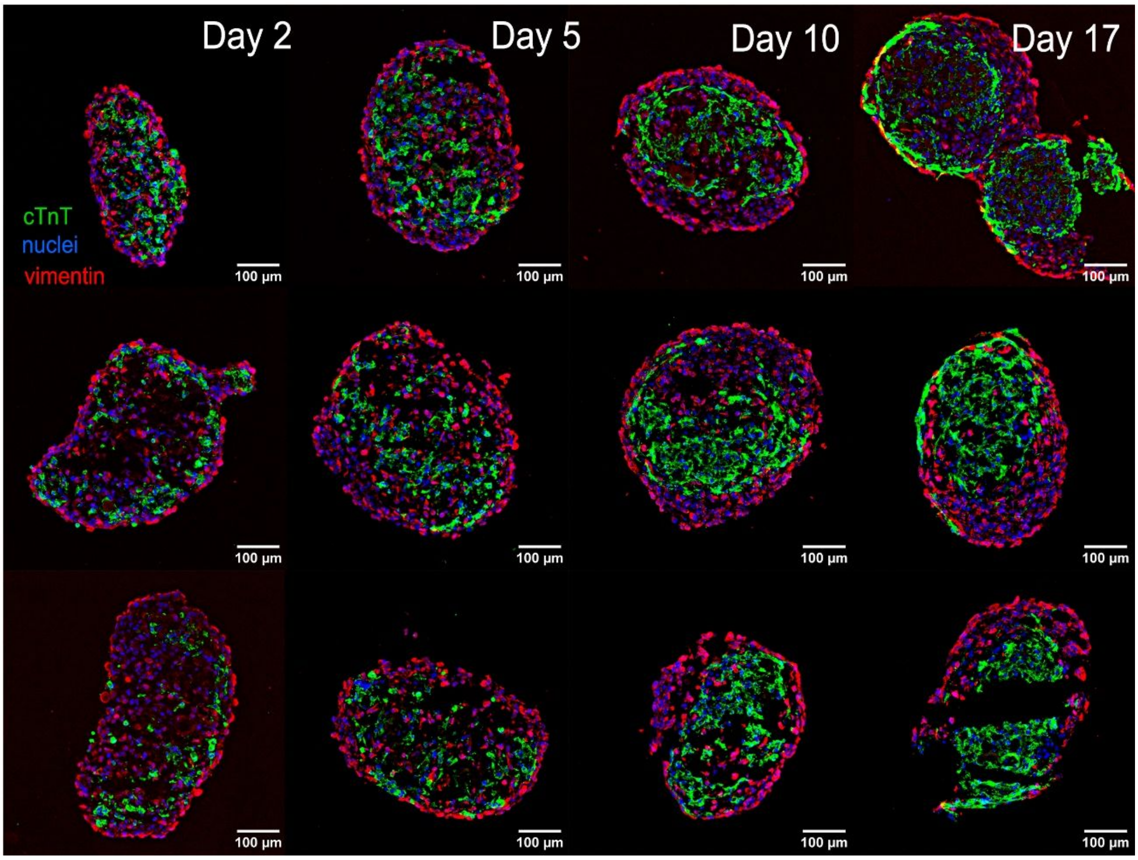

Figure S6. Troponin (cTnT, cardiomyocyte marker, in green) and vimentin (fibroblast marker, in red) distribution in cardiac spheroids cryosections at defined timepoints. Nuclei stained with Hoechst (blue).

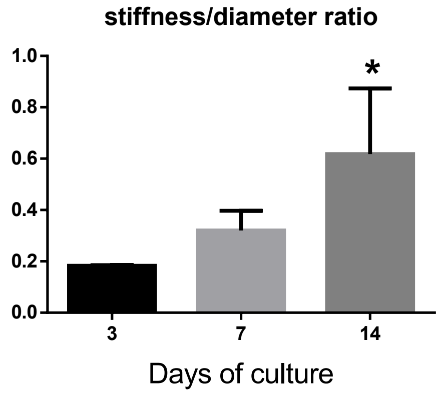

Figure S7. Ratio of spheroid’s stiffness/diameter investigated for each timepoint.

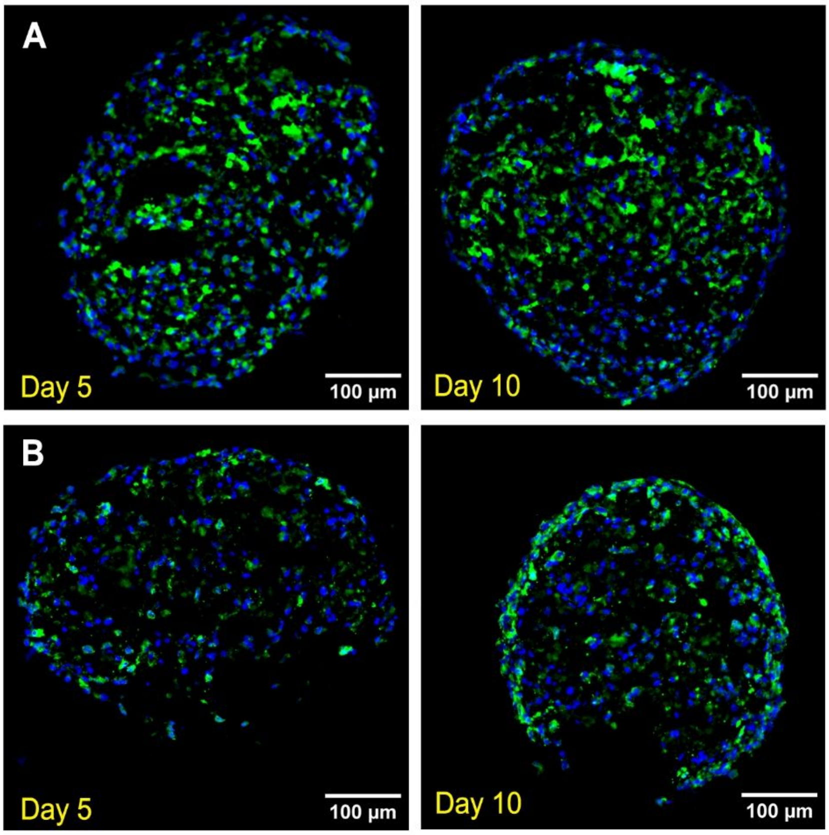

Figure S8. A) Collagen type I and B) collagen type III expression by immunohistofluorescence within spheroids at day 5 and day10. Blue: nucleus and Green: protein staining.
